# Fast, automated optimization of virtual monoenergetic images with the dual‐energy image synthesizer for cone‐beam CT

**DOI:** 10.1002/acm2.70083

**Published:** 2025-04-22

**Authors:** Andrew Keeler, Jason Luce, Mathias Lehmann, John C. Roeske, Hyejoo Kang

**Affiliations:** ^1^ Department of Radiation Oncology Stritch School of Medicine Cardinal Bernardin Cancer Center Loyola University of Chicago Maywood Illinois USA; ^2^ Varian Imaging Laboratory Baden Switzerland

**Keywords:** cone beam CT, dual‐energy, virtual monoenergetic images

## Abstract

**Background:**

Dual‐energy cone‐beam CT (DE‐CBCT) has become subject of recent interest due to the ability to produce virtual monoenergetic images (VMIs) with improved soft‐tissue contrast and reduced nonuniformity artifacts. However, efficient production and optimization of VMIs remains an under‐explored part of DE‐CBCT's application.

**Purpose:**

This work reports on the creation of DISC (dual‐energy image synthesizer for CBCT), a newly developed, open‐source user interface to efficiently produce and optimize VMIs with the eventual goal of clinical application.

**Methods:**

Two sets of CBCT scans of a Catphan 604 phantom were acquired sequentially (80 and 140 kVp) using the on‐board imager of a commercial linear accelerator. Material decomposition into aluminum (Al) and polymethyl‐methylacrylate (PMMA) basis materials in the projection‐domain and reconstruction with the Feldkamp–Davis–Kress (FDK) algorithm of basis material images were performed in the open‐source Tomographic Iterative GPU‐based REconstruction (TIGRE) Matlab toolkit. Using DISC, a series of VMIs were generated via linear combinations of the basis material images without reconstructing individual VMIs at different energies. Hounsfield units (HU) were computed using an energy‐dependent fit over the range of 20–150 keV. VMI energies that maximized contrast‐to‐noise ratio (CNR) for various materials and minimized nonuniformity artifacts were determined with 1 keV precision.

**Results:**

Optimal CNR values for all material inserts ranged from 55 to 62 keV, showing an average CNR enhancement of 25% over the polychromatic images. Optimal uniformity is observed at 65 keV. Computed HUs show good agreement with theoretical values, with root‐mean‐squared error of 16 HU across the range of energies and materials.

**Conclusion:**

A spectrum of VMIs from DE‐CBCT was efficiently produced with 1 keV precision using DISC. Optimal energies for both soft tissue contrast and nonuniformity reduction were quickly computed with high precision. Future work will expand DISC to generate other DE‐derived image types and will explore the acquisition and optimization of DE patient images.

## 1 INTRODUCTION

Cone‐beam CT (CBCT) is a commonly used imaging modality in image‐guided radiotherapy (IGRT). The flat‐panel imager can be directly mounted on the gantry of the treatment linac, facilitating easy patient position verification. CBCT also shows promise as a modality for online adaptive radiotherapy (ART),^1^, ^2^, ^3^, ^4^, ^5^ but CBCT images from conventional linacs generally show deteriorated soft‐tissue contrast and Hounsfield unit (HU) accuracy compared with the planning CT.^6^, ^7^, ^8^ This reduced CBCT image quality hinders registration of the CBCT and planning CT, which is a necessary part of current treatment practice.^9^

One proposed method to alleviate these concerns is the use of dual‐energy techniques in the CBCT acquisition (DE‐CBCT). DE‐CBCT can be used to create virtual monoenergetic images (VMIs) that can show enhanced soft tissue contrast and reduce nonuniformity artifacts caused by effects such as beam hardening.^10^ Artifact reduction may be beneficial for patient positioning during IGRT and segmentation in online ART. DE‐CBCT can also be used to directly create high accuracy relative electron density (RED) images without the need for HU‐RED conversion,^11^, ^12^ which could enhance the ability to perform accurate dose calculations and adaptations directly on the CBCT images. This feature may be highly beneficial for streamlining online ART workflows.^5^, ^13^

While recent work ^14^ has made DE‐CBCT available in the open‐source Tomographic Iterative GPU‐based REconstruction (TIGRE) toolkit, ^15^, ^16^ the predominant methods for CBCT VMI production ^10^, ^12^, ^14^, ^17^, ^18^ are time‐consuming and not practically suited for clinical workflows. Optimal VMI energies have been shown to vary for different treatment sites^19^, ^20^, ^21^, ^22^, ^23^ and may vary within a single image,^10^ making a priori knowledge of the ideal VMI energies difficult or impossible to achieve. Rapid online identification of the ideal VMI energies will be critical for accessing the improvements in contrast and uniformity over polychromatic CBCT images. Commonly, coarse energy intervals of 10 keV or more are used when generating ranges of VMIs^10^, ^12^, ^14^ that may not adequately locate these ideal energies for tissue contrast or artifact reduction. While finer energy spacings between VMIs would be preferable, the use of wider energy spacing in past works may be attributable to the computationally expensive process of repeated reconstructions to generate VMIs at various energies. This process is time‐consuming and would hinder the ability to perform the necessary online optimizations of VMIs.

We introduce DISC (dual‐energy image synthesizer for CBCT), a novel and user‐friendly open‐source interface to automate the generation and analysis of DE‐CBCT‐derived VMIs. To facilitate this development, we implement a computationally efficient VMI generation procedure. DISC leverages the improvements in speed to enable direct optimization of VMIs using a semicontinuous energy spectrum with 1 keV spacing, a factor of 10 finer than used in past DE‐CBCT works. To the best of our knowledge, our study is the first DE‐CBCT project to investigate optimizing VMI image quality with this energy precision.

## 2 METHODS

### 2.1 Dual‐energy imaging theory

The principle underlying dual‐energy (DE) CT and CBCT imaging was first described by Alvarez and Macovski.^24^ In short, the two primary contributions to x‐ray attenuation at the energies used for CT and CBCT imaging, the photoelectric effect and Compton scattering, are modeled using two representative materials with different relative cross sections for each interaction. These representative materials form a linearly independent basis of materials, so the attenuation characteristics of any other material at any energy can then be expressed as a linear combination of equivalent thicknesses of these basis materials.^25^

(1)

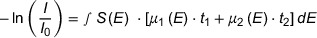

where *I_0_* and *I* represent the primary and attenuated x‐ray intensities from the kilovolt source, *S(E)* represents the normalized x‐ray spectrum, *µ_i_* represents the linear attenuation coefficient of the *i*th basis material, and *t_i_* represents the equivalent thickness of the *i*th basis material. Aluminum (Al) and PMMA are commonly chosen basis materials in the DE‐CBCT literature.^10^, ^11^, ^12^, ^14^, ^18^ Images at two different energy settings can be used to create a system of Equation (1) that can be solved for the relevant *t_i_*.^26^ This material decomposition procedure can be performed either in the projection domain, which is the approach used here, or in the reconstructed image domain.^27^, ^28^, ^29^ Once the equivalent thicknesses have been computed, virtual images can be synthesized with alternative x‐ray spectra *S(E)*, such as VMIs which set *S(E)* to describe a single‐energy beam of photons.

**FIGURE 1:**
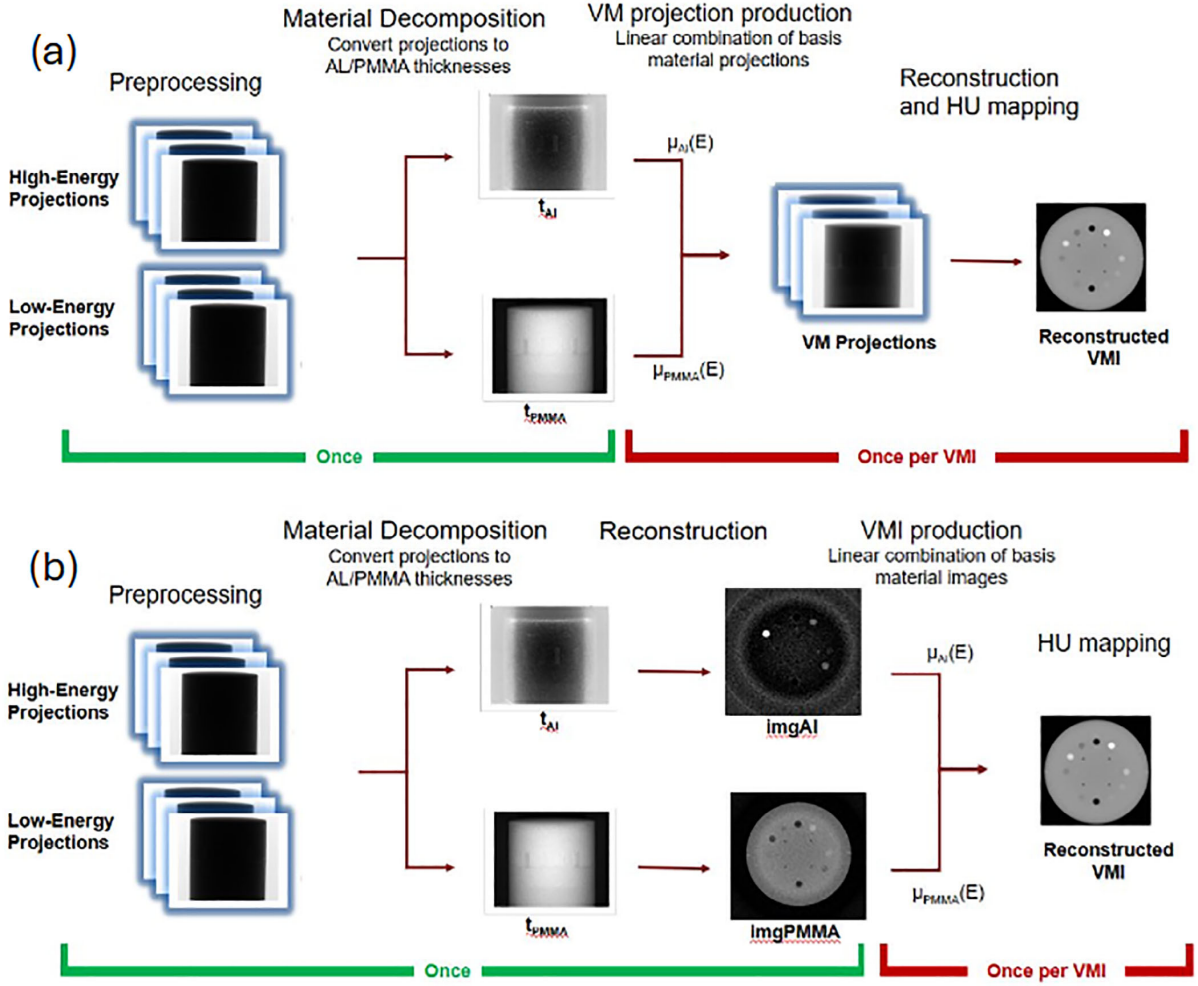
Comparison of two VMI production methods, using Al and PMMA basis materials. (a) shows the typical method seen in the DE‐CBCT literature,^10^, ^12^, ^14^, ^17^, ^18^ while (b) shows the proposed fast method adopted from DE‐CT.^11^, ^30^, ^31^, ^32^, ^33^, ^34^, ^35^ Due to the removal of the reconstruction step from the repeated portion of the procedure, the modified procedure can generate multiple VMIs much faster than the typical procedure. DE‐CBCT, dual‐energy cone‐beam CT; PMMA, polymethyl‐methylacrylate; VMIs, virtual monoenergetic images.

The typical VMI production method for projection‐domain DE‐CBCT^10^, ^12^, ^14^, ^17^, ^18^ is illustrated in Figure 1a. Following basis material decomposition, virtual monoenergetic projections (VMPs) are synthesized by taking a linear combination of the equivalent thickness projections with weights equal to the basis material attenuation coefficients at the desired energy. The set of VMPs can then be reconstructed into the VMI and mapped to HU. Since the VMI energy is determined at the VMP synthesis step, this procedure requires a new reconstruction procedure for each different VMI that would be generated, which becomes increasingly time consuming as the number of distinct VMIs increases.

An alternative method is illustrated in Figure 1b. This technique is common in DE‐CT^30^, ^31^, ^32^, ^33^, ^34^ but has not been widely implemented in the DE‐CBCT domain. In this procedure the equivalent thickness projections are reconstructed into basis material images where the voxel values represent the relative fraction of each basis material corresponding to the attenuation properties of the object material in that voxel. VMIs at different energies can then be directly synthesized by taking linear combinations of the basis images using the same attenuation coefficient weights as used when synthesizing VMPs. Here the VMI energy is determined after the reconstruction of the basis images, so producing additional VMIs requires only a simple linear combination operation and can be carried out orders of magnitude more quickly. We refer to this as the fast production method.

### 2.2 DISC

**FIGURE 2:**
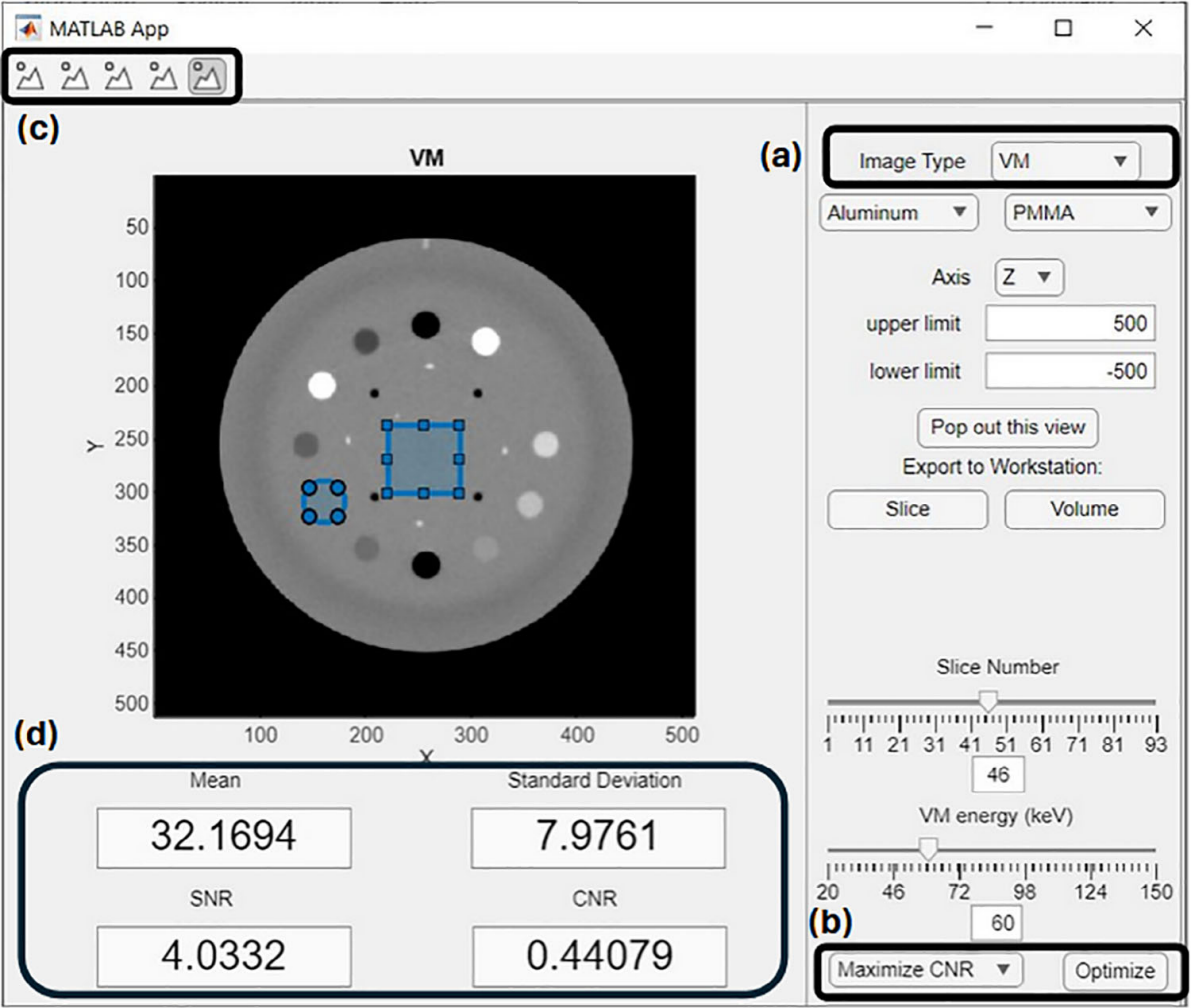
DISC UI displaying a VMI synthesized at 60 keV. (a) The UI is designed to allow users to quickly access the polychromatic scans, basis material images, and VMIs at arbitrary energies via the dropdown menu at the top‐right of the interface. (b) Preprogramed optimization procedures can be set from the dropdown on the bottom right. (c) ROIs can be drawn by selecting the desired geometry from the buttons on the top‐left, with (d) average pixel values, standard deviation of pixel value, and SNR over the ROI being automatically computed and displayed below the image. When two ROIs are drawn, CNR between the two ROIs is also computed. CNR, contrast‐to‐noise ratio; DISC, dual‐energy image synthesizer; ROIs, regions of interest; SNR, signal‐to‐noise ratio; UI, user interface; VMI, virtual monoenergetic image.

The newly‐developed DISC is an open‐source, Matlab‐based user interface (UI) to facilitate this fast production of VMIs. DISC's UI, depicted in Figure 2, accepts as inputs the two reconstructed polychromatic CBCT images and the two corresponding reconstructed basis material images. It then allows the user to quickly compute VMIs from the basis images. VMIs can be generated at energies ranging from 20 to 150 keV using material‐specific attenuation values for the basis materials taken from the NIST XCOM database^36^ in 10‐keV increments. A VMI energy resolution of 1 keV is achieved by using a cubic spline interpolation of the basis material attenuation values. VMI energies can be manually selected using either a slider or input field.

The HU scale is energy‐dependent, so typically each VMI energy would require its own calibration function to convert the linear attenuation pixel values to the corresponding HU. Instead, DISC uses a single, energy‐dependent fit function to interpolate the HU at arbitrary energies. This function has a linear dependence on attenuation coefficient value, as the typical HU scale does, but also incorporates a 4th‐degree energy dependence to model the effect of varying the VMI energy on each material's attenuation coefficients. To calibrate this fit, HU are computed for the material inserts in a Catphan 604 phantom at 20, 30, 40, 50, 60, 80, 100, and 150 keV using the attenuation coefficients published by the manufacturer (The Phantom Laboratory, Salem, NY). These HU are then fit to their corresponding pixel values at the appropriate energy using a least‐squares regression model.

Up to two regions of interest (ROIs) can be drawn on the displayed image in several geometries, including circular, polygon, and freehand. Once drawn, DISC will automatically compute and display the mean pixel value, pixel value standard deviation, and signal‐to‐noise ratio (SNR) for the active ROI. Once a second ROI is drawn, the contrast‐to‐noise ratio (CNR) between the two active ROIs is also computed and displayed. Both ROIs are propagated to the active image as it is updated, and drawing additional ROIs sequentially replaces the old ROIs in the drawing order so that the maximum number of active ROIs remains two (see Figure 2).

DISC contains several preprogramed optimization procedures for VMIs. VMIs of optimized energies are expected to show enhanced CNR or reduced nonuniformity effects such as those caused by beam hardening or metal. Users can select two ROIs and then ask DISC to locate and display the optimal VMI energy for either CNR maximization or contrast minimization between the two ROIs. Additionally, for the Catphan 604 phantom, the clinical uniformity check, consisting of five pre‐set circular ROIs of radius 26 pixels at the top, bottom, left, right, and center of the uniformity module of the phantom is preprogramed and can be run by selecting the appropriate slice of the CBCT image. Upon completion, each of these optimization procedures displays the optimal VMI in the display pane and shows a window plotting the selected metric against the VMI energy for the entire energy range so users can assess the optimization results directly.

### 2.3 Data acquisition

To evaluate the performance of the VMI production process as implemented by DISC, data was taken of a Catphan 604 phantom using the on‐board imager of a Varian TrueBeam linac (Siemens Healthineers). Two sequential scans were acquired, one at 80 kV, 20 mA, 60 ms, and the other at 140 kV, 20 mA, 10 ms. Each scan was acquired using a full‐fan, full trajectory protocol (field‐of‐view = 25 cm) at 11 frames per second with the bowtie filter in place, resulting in approximately 660 projections at each voltage setting. These acquisition settings have been used previously in DE‐CBCT^12^, ^14^ and provide an approximately equal dose partition between the high‐ and low‐kilovolt scans. The acquired projections were decomposed into equivalent thickness projections of basis materials Al and PMMA using the open‐source TIGRE‐DE toolkit.^14^ Due to the small angular separation between consecutive projections, a nearest‐neighbor sinogram interpolation scheme was used to match the gantry angle between the low‐ and high‐kilovolt projection sets.

### 2.4 Data analysis

Following decomposition into equivalent thickness projections of basis materials, the two VMI production procedures, typical (Figure 1a) and fast (Figure 1b), were compared for their speed at producing a set of 131 VMIs with energies ranging from 20 to 150 keV in 1 keV increments. For each process, the evaluation encompassed the reconstruction and linear combination steps, including the interpolation of the appropriate material attenuation coefficients, of the respective VMI production procedures, plus a plotting step to mirror as closely as possible the optimization procedure developed within DISC. Reconstruction was performed using the FDK algorithm included with the TIGRE toolkit, which uses GPU parallelization to solve the reconstruction problem in a fast, memory‐efficient manner. A Ram‐Lak filter was used as the reconstruction kernel and images were produced with a voxel size of 0.5 mm × 0.5 mm × 2 mm. The comparison test was carried out using a desktop computer system running Matlab 2024a on a Windows 11 operating system with an Intel i5 vPRO CPU and an Nvidia GTX 1050Ti GPU. Each procedure was evaluated based on the time needed to complete the first and second VMI syntheses, the average marginal time cost to produce the third and subsequent VMIs, and the total elapsed time to synthesize the entire set of VMIs.

VMI image quality was assessed using the metrics of HU consistency, optimal CNR within the VMI image set, and optimal uniformity within the VMI image set. To assess HU consistency, HU computed for the various material inserts were compared to the nominal HU values for those inserts at 40, 50, 60, 80, 100, and 150 keV. This same comparison was carried out in the development of the TIGRE‐DE toolkit,^14^ allowing for a direct comparison between the HU mapping method in this work compared with past work.

CNR is computed as

(2) $$\mathrm{CN}R_{i}=\frac{|HU_{i}-HU_{\mathrm{bkg}}|}{\sqrt{\frac{1}{2}\cdot \left(\sigma_{i}^{2}+\sigma_{\mathrm{bkg}}^{2}\right)}}\;$$

where the subscript *i* refers to the material insert under consideration, *bkg* refers to the background material of the phantom, *HU* indicates the average HU over the corresponding circular ROI, and *σ* indicates the standard deviation of the HU value over the corresponding ROI.

Image uniformity is assessed using the automated uniformity test coded into DISC. Nonuniformity is computed as the largest absolute difference between the average HU value of all five ROIs and the average HU of each of those ROIs individually.

## 3 RESULTS

### 3.1 VMI production efficiency comparison

In the comparison test, the fast DE‐CBCT procedure generates new VMIs almost three orders of magnitude faster than the typical procedure. If the upfront time cost of the two basis image reconstructions is considered, the fast procedure remains more efficient at producing multiple VMIs, with an overall efficiency gain of almost two orders of magnitude for producing the entire VMI energy range of 131 VMIs. The specific results of the timing test can be found in Table 1.

**TABLE 1:** Comparison of timings between the typical and fast VMI generation procedure (see Figure 1).

Procedure	1st VMI (s)	2nd VMI (s)	Additional VMIs (s)	Total time for 131 VMIs (s)
Typical	19.2	17.6	17.4	2276.1
Fast	34.0	0.02	0.02	36.5

*Note*: Computations were made in Matlab 2024a running on Windows 11 operating system, Intel i5 vPRO CPU and Nvidia GTX 1050Ti GPU. The reconstruction process was performed using the TIGRE toolkit.

Abbreviation: VMIs, virtual monoenergetic images.

### 3.2 HU consistency comparison

**FIGURE 3:**
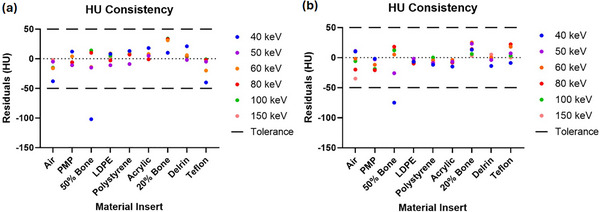
HU consistency. (a) shows the HU errors plotted from TIGRE‐DE, which used several energy‐specific HU calibrations.^14^ (b) shows the corresponding plot for HU computed using DISC, which uses a single, energy‐dependent fit. Both the largest errors and the overall RMSE show slight improvements in (b) compared with (a). HU, Hounsfield units; RMSE, root‐mean‐squared errors.

The consistency of the energy‐dependent HU calibration can be seen in Figure 3. In a direct comparison with the HU mappings published alongside the TIGRE‐DE toolkit,^14^ modest improvements in HU consistency are observed using DISC, with the overall root‐mean‐squared errors (RMSE) of the HU values improving from 20.2 to 16.2 HU. The improved error values can be largely attributed to the reduction in the large deviations from theoretical HU observed in the 50% bone and Teflon inserts at 40 keV, with the error in the 50% bone HU falling from 102 HU observed in previous work to 75 HU in this study and the error in the Teflon insert similarly falling from 40 to 9 HU.^14^ Overall, the energy dependent HU calibration shows at least comparable agreement with expected HU values compared to the ad hoc fits used previously.^12^, ^14^ Overall, the HU RMSE of 16 HU would represent a shift of only a few kiloelectron volt in the optimal VMI energies, so the optimal energies computed in the following sections should be robust against errors in HU values.

### 3.3 VMI energy optimization

**FIGURE 4:**
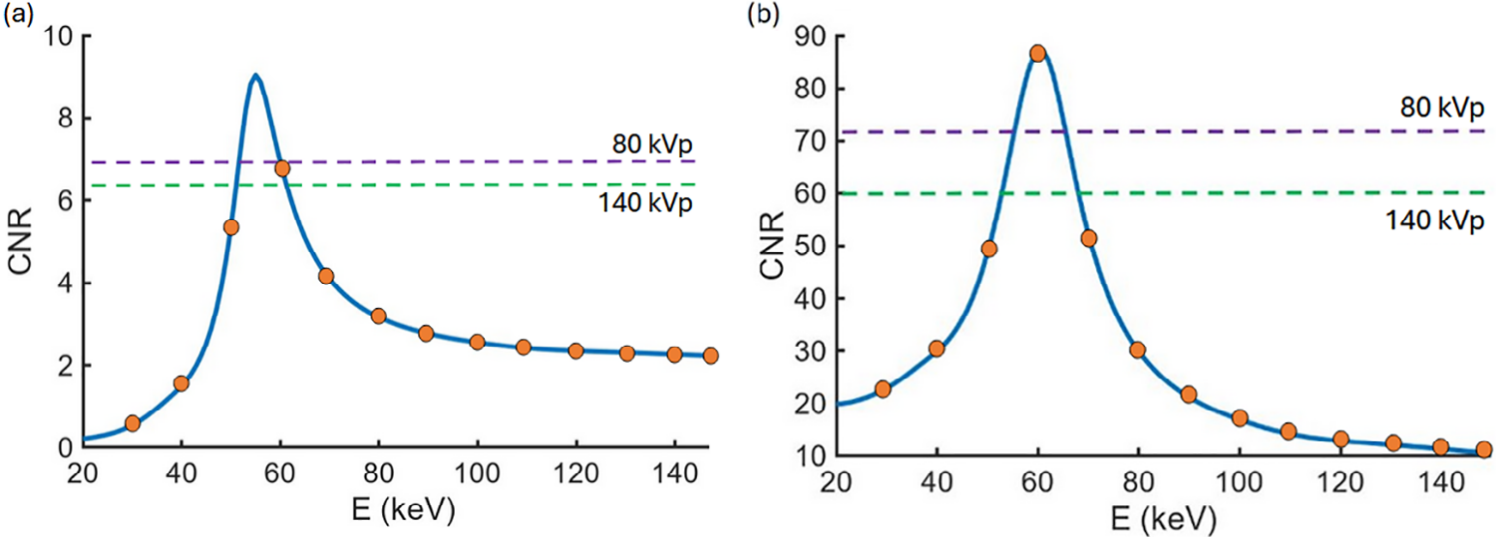
CNR as a function of VMI energy for (a) the low‐contrast acrylic insert and (b) the high‐contrast 50% bone insert. The optimal VMI energies observed are (a) 55 keV and (b) 61 keV. In each case, the CNR peak is relatively narrow, so coarse VMI energy spacings of 10 keV (indicated by the orange markers) may miss the optimal values. The corresponding CNR from the polychromatic CBCT scans are indicated by the horizontal dashed lines. In both cases, selection of the optimal VMI energy produces a higher CNR value than either of the DE image pairs alone. CNRs for the other material inserts in the phantom show similar behavior. CBCT, cone‐beam CT; CNR, contrast‐to‐noise ratio; VMI, virtual monoenergetic image.

**FIGURE 5:**
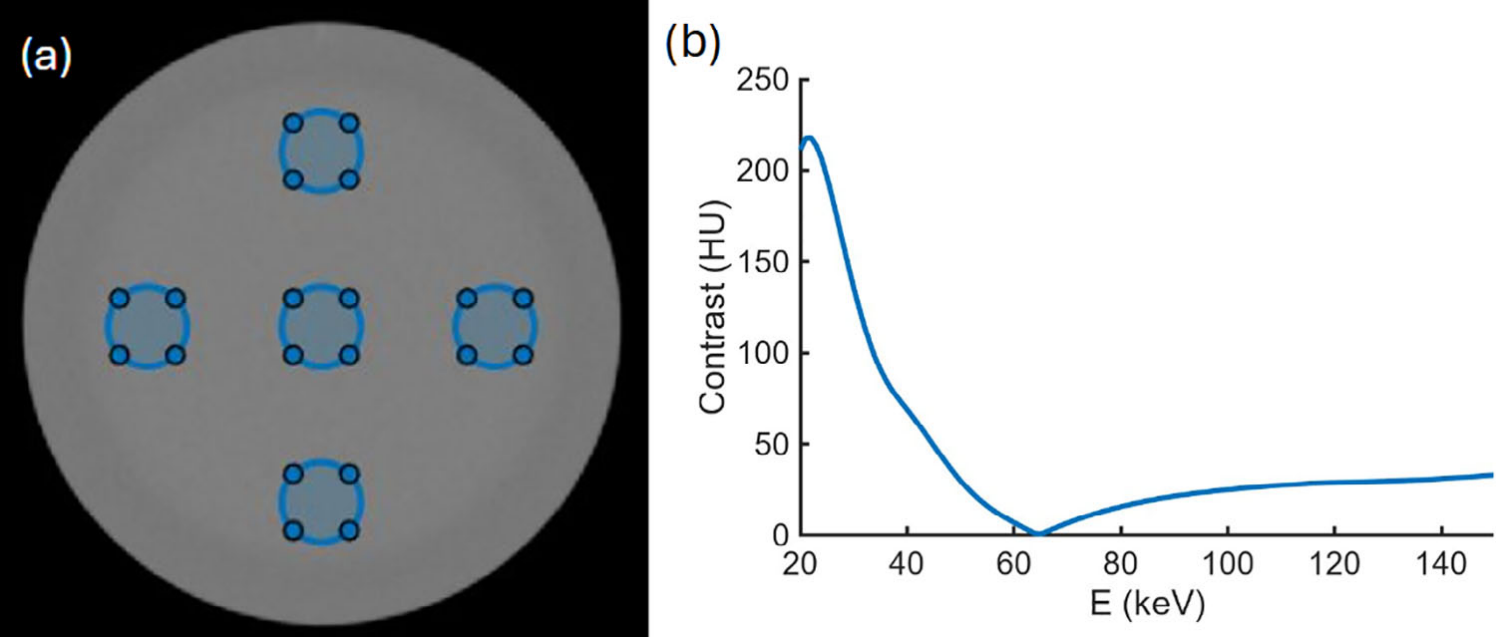
Evaluation of the uniformity module in the Catphan 604. (a) shows the five ROIs used to perform the test, which mirrors the uniformity test used for clinical quality assurance. (b) shows the results of the uniformity test as a function of VMI energy. Nonuniformity is evaluated as the largest deviation of an ROI's HU from the average HU of all five ROIs. The minimal nonuniformity of < 1 HU is found at 65 keV. Accounting for the noise level of ∼8 HU, the energy range of 60–72 keV is consistent with minimal nonuniformity. HU, Hounsfield units; ROIs, regions of interest; VMIs, virtual monoenergetic images.

Figures 4 and 5 show examples of typical results of VMI optimization procedures for CNR and uniformity, respectively, using DISC. For optimizing CNR of the Catphan 604 material inserts against the background material in 1 keV increments, optimal VMI energies range from 55 to 62 keV depending on the material insert being assessed, with the optimal VMIs showing systematic enhancements of CNR over the polychromatic images. CNR enhancements for the material inserts ranged from 15% to 36% over the polychromatic image with the largest CNR for that insert, with the average enhancement being 25% over the clearer of the two polychromatic images. Optimal uniformity of < 1 HU deviation from the average is observed at 65 keV, and a range of energies from 60 to 72 keV are all consistent with zero nonuniformity up to the ∼8 HU noise level (Figure 5).

Optimizing VMIs to 1 keV precision improves the ability to accurately identify the VMI energy of peak CNR or minimal nonuniformity compared with the larger 10 keV spacing employed in other DE‐CBCT studies. In the case of the acrylic insert (Figure 4a), the optimal VMI energy of 55 keV means that the 10 keV spacing, which produces VMIs at 50 and 60 keV, does not produce a CNR greater than that from the 80 kVp polychromatic scan. Hence, the true benefit of the VMI would not be observed for this material, even though the 50% bone insert from the same image does have an optimal energy that is well‐located by the wider energy spacing (Figure 4b). Utilizing the fast VMI procedure to perform this analysis allows these optimization procedures to be performed and the results displayed in less than 15 s compared to 40 min that would be required using the typical VMI procedure.

## 4 DISCUSSION

This work is the first to directly explore the optimization of VMIs derived from DE‐CBCT imaging to 1 keV energy precision by utilizing a fast procedure for VMI generation. While past works have explored the creation of optimized VMIs in the both the DE‐CBCT and DE‐CT literature,^10^, ^12^, ^14^, ^18^, ^19^, ^20^, ^21^, ^22^, ^23^ they generally opt for coarse energy intervals of 10 keV or larger, hindering the ability to accurately identify the optimal energies for either CNR enhancement or artifact reduction.

The majority of the existing DE‐CBCT literature is focused on exploring methods for acquisition of DE‐CBCT using methods such as fast kilovolt switching,^12^ multisource acquisition,^10^ split and rotating filters,^37^ dual‐layer detectors^35^ or sequential scans.^11^, ^18^ Some of these works consider the generation of other DE‐derived image types such as RED^11^ or blended images,^37^ but not VMIs. Of those works that deal with VMIs, Li,^10^ Cassetta,^12^and Skaarup,^18^ provide only coarse optimization for CNR, with only Li considering additional optimization around image uniformity.

Other studies focus on the method of material decomposition rather than the data acquisition method. In addition to those mentioned above, Keeler^14^ makes available a simple, projection‐domain decomposition algorithm in an open‐source context, but like the work by Cassetta^12^ that it is based on, uses only coarse energy intervals for its VMIs. Several works^27^, ^28^, ^29^ employ image‐domain material decomposition, but likewise only coarsely optimize the VMI energies with respect to CNR and cannot optimize image uniformity due to the inherent limitations of the image‐domain material decomposition method. Wang et al.^35^ proposes a more complex hybrid material decomposition scheme but makes no mention of optimizing the VMIs themselves and only shows a single VMI energy.

By comparison, this work's focus on the process of VMI creation and optimization emphasizes the importance of VMI generation and analysis in a fast, computationally efficient manner. Prospective clinical applications of DE‐CBCT for IGRT and ART would require the quick production, review, and optimization of VMIs and other DE‐derived image types such as RED,^11^, ^12^ and will need to be flexible enough to accommodate the various potential ways such as workflow can be implemented. The development of DISC facilitates this in three ways. First, utilizing the fast DE‐CBCT procedure with interpolated attenuation coefficients and HU mapping allows for easy creation of and efficient switching between VMIs with low memory overhead. This in turn enables a much finer energy resolution of 1 keV for the VMIs here compared with the larger intervals seen elsewhere in the DE‐CBCT literature.^10^, ^12^, ^14^, ^18^ Second, by employing a high degree of automation, DISC can be made much more user‐friendly, requiring less technical knowledge to create and analyze the desired images. Automation of the VMI optimization process is especially important given that both past work^10^, ^19^, ^20^, ^21^, ^22^, ^23^ and this work show clearly that the optimal VMI energy can vary within an image or between images. This dependence of the optimal VMI energy on the material or site being visualized, or the desire for improved uniformity versus CNR enhancement, will naturally limit the ability of prior knowledge to compensate for direct optimization of VMIs. Third, DISC is agnostic regarding the modes of data acquisition and decomposition. As such, DISC would be broadly applicable to developing DE‐CBCT workflows that may use other acquisition and decomposition methods than those employed here.

This work also illustrates the importance of fine energy resolution in accurately identifying VMI energies with optimal characteristics compared with the coarse intervals used in past works^10^, ^12^, ^14^, ^18^ that can provide at best rough approximations of optimal energies for VMIs. Due to the observed narrowness of the CNR distribution as a function of VMI energy, the VMI energy displaying optimal CNR may not be well identified by a 10 keV energy spacing, as shown in Figure 4a. Further, the optimal VMI energy shows variation between different materials and treatment sites,^10^, ^19^, ^20^, ^21^, ^22^, ^23^ so the ability to quickly compute the optimal VMI energy with this fine energy spacing would be essential. Past work in DE‐CT has similarly found that optimal VMI energies show similar narrow energy ranges of CNR enhancement.^19^, ^21^ While identifying the mechanism behind the narrowness of the CNR peaks would be a promising avenue for future investigation, it is beyond the scope of this work.

Investigations in DE‐CT imaging have shown that one of the major factors affecting optimal VMI energies with respect to CNR is the underlying noise level in the image and that optimal energies tend to be those that minimize the noise.^19^, ^21^ While noise reduction techniques beyond optimal VMI energy selection are not employed in this work, the image volumes constructed with DISC can be exported to the Matlab workspace at the push of a button to further refine using postprocessing noise reduction techniques. Future work using DISC can explore the incorporation of more robust noise reduction techniques both preprocess as part of the reconstruction of the basis material images and in‐process during the image synthesis and energy optimization procedures.

Relatedly, the scan acquisition settings used in here were chosen to match those used in previous DE‐CBCT studies,^12^, ^14^ While this choice provides direct comparisons between this study and the previous studies, the effects of various dose partitioning schemes remain a subject of ongoing research.

While currently DISC only provides access to VMIs, the open‐source implementation of the platform makes the incorporation of additional DE‐derived image types obvious avenues for future work. In particular, directly computed RED images have shown promise in previous DE‐CBCT studies.^11^, ^12^ Incorporation of RED images into DISC could be of great benefit given the desired application of DE‐CBCT to online ART that this work hopes to facilitate. Additionally, DISC is easily modifiable to handle other basis material combinations such as Iodine and water in addition to the Al and PMMA basis used in this work. Future extensions of the platform can include handling of these alternate basis material combinations and potentially consider computation of three‐material bases such as Iodine/water/fat that can be used to create material maps and synthesize virtual non‐contrast images.^38^ Finally, studies using more complex anthropomorphic phantoms and patient data would provide further tests of the robustness of the fast VMI synthesis procedure and optimization procedures incorporated into DISC, as well as the potential impacts of DE‐CBCT on clinical decision‐making.

## 5 CONCLUSION

This work presents the development of DISC, an accessible, open‐source platform for the fast creation of and easy, automated analysis of DE‐CBCT VMIs. We successfully validated the performance of the fast DE‐CBCT VMI procedure with DISC, enabling the quick, accurate optimization of VMI energies based on CNR and uniformity metrics with fine energy spacing. Future work includes expanding the variety of DE‐CBCT image types available within the platform and using the platform to assess the performance of DE‐CBCT in enhancing the image quality of patient data. This work paves a way towards the use of DE‐CBCT within clinically feasible timeframes. The DISC UI is available at https://github.com/akeeler/DISC.

## AUTHOR CONTRIBUTIONS

**Andrew Keeler**: Research Conceptualization; Methodology contribution; manuscript preparation. **Jason Luce**: Methodology contribution; manuscript preparation. **Mathias Lehmann**: Methodology contribution; manuscript preparation. **John C. Roeske**: Methodology contribution; manuscript preparation. **Hyejoo Kang**: Methodology contribution; manuscript preparation.

## CONFLICT OF INTEREST STATEMENT

M.L. is an employee of Varian Medical Systems.
